# The Lipid Phenotype of Breast Cancer Cells Characterized by Raman Microspectroscopy: Towards a Stratification of Malignancy

**DOI:** 10.1371/journal.pone.0046456

**Published:** 2012-10-17

**Authors:** Claudia Nieva, Monica Marro, Naiara Santana-Codina, Satish Rao, Dmitri Petrov, Angels Sierra

**Affiliations:** 1 IDIBELL-Institut d'Investigació Biomèdica de Bellvitge, L'Hospitalet de Llobregat, Barcelona, Spain; 2 ICFO-Institut de Ciències Fotòniques, Parc Mediterrani de la Tecnologia, Castelldefels, Barcelona, Spain; 3 UAB-Universitat Autònoma de Barcelona, Campus Bellaterra, Cerdanyola del Vallés, Barcelona, Spain; 4 Mount Sinai School of Medicine, New York, New York, United States of America; 5 ICREA-Institució Catalana de Recerca i Estudis Avançats, Barcelona, Spain; Wageningen University, The Netherlands

## Abstract

Although molecular classification brings interesting insights into breast cancer taxonomy, its implementation in daily clinical care is questionable because of its expense and the information supplied in a single sample allocation is not sufficiently reliable. New approaches, based on a panel of small molecules derived from the global or targeted analysis of metabolic profiles of cells, have found a correlation between activation of *de novo* lipogenesis and poorer prognosis and shorter disease-free survival for many tumors. We hypothesized that the lipid content of breast cancer cells might be a useful indirect measure of a variety of functions coupled to breast cancer progression. Raman microspectroscopy was used to characterize metabolism of breast cancer cells with different degrees of malignancy. Raman spectra from MDA-MB-435, MDA-MB-468, MDA-MB-231, SKBR3, MCF7 and MCF10A cells were acquired with an InVia Raman microscope (Renishaw) with a backscattered configuration. We used Principal Component Analysis and Partial Least Squares Discriminant Analyses to assess the different profiling of the lipid composition of breast cancer cells. Characteristic bands related to lipid content were found at 3014, 2935, 2890 and 2845 cm^−1^, and related to lipid and protein content at 2940 cm^−1^. A classificatory model was generated which segregated metastatic cells and non-metastatic cells without basal-like phenotype with a sensitivity of 90% and a specificity of 82.1%. Moreover, expression of SREBP-1c and ABCA1 genes validated the assignation of the lipid phenotype of breast cancer cells. Indeed, changes in fatty acid unsaturation were related with the epithelial-to-mesenchymal transition phenotype. Raman microspectroscopy is a promising technique for characterizing and classifying the malignant phenotype of breast cancer cells on the basis of their lipid profiling. The algorithm for the discrimination of metastatic ability is a first step towards stratifying breast cancer cells using this rapid and reagent-free tool.

## Introduction

Despite the reduction in mortality in breast cancer patients due to earlier diagnosis and implementation of adjuvant chemo- and hormone therapies, breast cancer is still the commonest cause of cancer death in women worldwide [1]. Many factors and genes are involved in the initiation of breast cancer, but mortality is due to metastatic disease [2]. Patients who go on to develop life-threatening metastases in the visceral tissues have a much higher mortality rate and shortened life expectancy [3], [4].

Although the different biological behaviors and metastatic patterns observed among the distinct breast cancer phenotypes may suggest different mechanisms of invasion and metastasis, the biological features of breast tumors have proven insufficient for a comprehensive description of progression at first diagnosis, due to the heterogeneity of the disease [5]. The datasets available use specific genomic alterations to define subtypes of breast cancer [6]. However, the large number of genetic alterations present in tumor cells complicates the discrimination between genes that are critical for maintaining the disease state and those that are merely coincidental [7]. Thus, although molecular classification provides interesting insights into breast cancer taxonomy, its implementation in clinical care is questionable because it is too expensive to be introduced in daily pathological diagnosis, and because the information supplied is of insufficient reliability in single sample allocation [8].

Many observations during the early period of cancer biology research identified metabolic changes as common features of cancerous tissue, such as the Warburg effect [9], [10]. New approaches based on a panel of small molecules derived from the global or targeted analysis of metabolic profiles of cells are being developed to link cancer and altered metabolisms and to characterize cancer cell–specific metabolisms [11], [12]. One of the clearest signals is the *de novo* production of fatty acids in tumor cells associated with cancer progression, linked to an increased need for membranes during rapid cell proliferation as a part of a more general metabolic transformation, which provides cancer cells with autonomy in terms of their supply of building blocks for growth [13]. This metabolic change occurs as a result of common oncogenic insults and is mediated by the activation of multiple lipogenic enzymes affected at all levels of regulation, including transcription, translation, protein stabilization and protein phosphorylation [14]–[16]. Activation of *de novo* lipogenesis correlated with a poorer prognosis and shorter disease-free survival for many tumor types [17], [18]. A low ratio of TUFA/TFA has been proposed as a molecular marker for these aggressive tumors, which is called the lipogenic phenotype. The pathway that regulates synthesis of fatty acid in normal and tumor cells shares identical downstream elements including the SREBP-1c (transcriptional regulator sterol regulatory element-binding protein-1) and LXR (liver X receptor) [15], [19]. We hypothesized that the lipid content of breast cancer cells might be an indirect measure of a variety of functions coupled to breast cancer progression, and that it could discriminate between different genetic features of breast cancer cells, providing new information on the aggressiveness of their phenotype.

To explore the lipid phenotype associated with breast cancer malignancy we used Raman microspectroscopy (RS). RS is an optical technique that utilizes molecular-specific, inelastic scattering of photons to interrogate biological material [20]. When a sample is illuminated with an optical beam, a small fraction of the photons is inelastically scattered by the intramolecular bonds present. When this occurs, the photon donates energy to, or receives energy from, the molecule, producing a change in the molecule's vibrational state. When it subsequently exits the material, the photon has an altered energy level and, therefore, an altered wavelength. This change in the photon's energy is known as the ‘Raman shift’ and is measured in wavenumbers. Photons interacting with different biochemical bonds undergo specific Raman shifts, which, considered together, form the ‘Raman spectrum’, a plot of intensity against the Raman shift and a direct function of the molecular composition of the material studied. When applied to biological tissue, the technique can distinguish between pathologies based on the differences in their biochemical makeup [21]. RS is a rapid, reagent-free and non-destructive alternative for the analysis of cell biology systems [22]. Recent advances in Raman spectroscopy have given way to a wide range of biomedical applications including cancer. Its ability to detect variance related to DNA/RNA, proteins, and lipids have made it an excellent tool for quantifying changes on the cellular level, as well as differentiating between various cell fingerprints over all the Raman spectral range. The collection of spectra can be performed *in vitro, ex vivo or in vivo* without disrupting the cellular environment [23]. This is a major advantage of Raman spectroscopy, as most biological assays utilize chemical biomarkers and often require conditions nonnative to the biological environment.

Usually, Raman spectra of biological samples are highly complex, and so mathematical processing of the spectroscopic data is required to obtain objective information. Multivariate techniques reduce the dimensionality of the spectral data and allow extraction of useful, objective and less complex information [24], [25]. We used Principal Component Analysis (PCA) [26] and Partial Least Squares Discriminant Analyses (PLS-DA) [27] to assess the different profiling of the lipid composition of breast cancer cells, which permitted differentiation of the lipogenic phenotype according to the proportion of unsaturated fatty acids. Moreover, PCA clearly distinguished cells with the epithelial-to-mesenchymal transition (EMT) phenotype, which is widely linked with breast cancer cell aggressiveness [28]. A discriminative model was generated that segregates metastatic cells and non-metastatic cells without basal-like phenotype with 90% sensitivity and 82.1% specificity.

## Materials and Methods

### Cell culture and treatments

MDA-MB-435, MDA-MB-468, MDA-MB-321, SKBR3, MCF7 and MCF10A cells were obtained from the American Type Culture Collection. With the exception of MCF10A, all lines were maintained under standard conditions in 1∶1 (v/v) mixture of DMEM and Ham F12 medium (DMEM/F12) supplemented with 10% fetal bovine serum (FBS), 1 mM pyruvate and 2 mM L-glutamine in 5% CO2-95% air at 37°C in a humidified incubator. MCF7 medium was supplemented with 0.01 mg/ml bovine insulin. MCF10A was grown in DMEM/F12 medium supplemented with 5% horse serum, 1 mM pyruvate, 2 mM L-glutamine, 0.01 mg/ml bovine insulin, 20 ng/ml EGF, 1 mg/ml hydrocortisone and 100 ng/ml Tetanus toxine, in the same incubator conditions described above. The treatment with the LXR agonist T0901317 (Cayman Chemical Company, Michigan), dissolved in DMSO, was performed at 2 µM final concentration (controls were treated with DMSO at the same concentration).

### Immunocytochemistry and labelling of cells

For immunocytochemistry 8×10^4^ cells were seeded in 24 well-plates containing cover slips and were fixed after 24 h using cold methanol for 1 min. MCF10A in sparse conditions was obtained with 8×10^3^ cells/well. Cells were washed three times with PBS1x and treated with PBS1x-5% FBS for 30 min at room temperature. The antibodies used were: Vimentin, mouse anti-human (Dako, Atlanta); E-cadherin, mouse anti-human (BD Biosciences, NJ). Antibodies were diluted 1∶50 in PBS1x-1% FBS and used for 1 h at room temperature. After three washes with PBS1x the secondary antibody, Alexa 555 anti mouse IgG (Life technologies, NY) was used diluted 1∶1000 in PBS1x-1% FBS for 30 min at room temperature. After three washes with PBS1x the cover slips were mounted on slides using Vectashield (Vector laboratories, Burlingame) with DAPI for nucleus visualization. Preparations were analyzed with an Olympus BX60 fluorescence microscope (Olympus, Japan), using the optimal filters and 40× magnification.

For Nile Red and filipin staining 8×10^4^ cells were seeded in 24 well-plates containing coverslips and 24 h later cells were fixed with 4% cold paraformaldehyde (PFA) in PBS1x for 15 min. After fixing, cells were washed three times with PBS1x and stained with Nile Red at a final concentration of 1 µg/ml for 1 h, or filipin at a final concentration of 50 µg/ml for 2 h. Coverslips were then mounted as described above (filipin staining without DAPI) and analyzed with the confocal microscope (Leica TCS SP5, Wetzlar, Germany) for Nile red and the Olympus BX60 fluorescence microscope for filipin, with 40× magnification.

### Raman spectroscopy

For analysis, 3×10^5^ cells were used, and for MCF10A cells in sparse conditions 3×10^4^ cells were used. For measurements in the 2820–3030 cm^−1^ range, cells were seeded in Petri dishes with #0 coverglass (Mattek, Ashland, MA). After 24 h, cells were treated as indicated for Nile red staining.

The Raman system Renishaw (Apply Innovation, Gloucestershire, UK) comprises a 514 nm laser that supplies an excitation beam of about 10 mW power, which is focused onto the sample via a microscope with 60× objective (Edmund, York, UK). The same objective collects the scattered light from the sample and directs it to the spectrometer. The spectrometer processes this scattered light, by rejecting the unwanted portion and separating the remainder into its constituent wavelengths. The Raman spectrum is recorded on a deep depletion charge-coupled device (CCD) detector (Renishaw RenCam). The recorded Raman spectrum is digitized and displayed on a personal computer using Renishaw WiRE software which allows the experimental parameters to be set.

The spectra were background subtracted with a custom-written Labview program and the Gaussian fits for total fatty acids (TFA) and total unsaturated fatty acids (TUFA) bands (2845 cm^−1^ and 3015 cm^−1^ respectively) were performed in Matlab allowing the quantification of the two types of fatty acids in the cytoplasm [20].

### Statistical analysis

Raman spectroscopy is a promising technique in biomedical studies due to its non-invasive character and high specificity but Micro-Raman spectra of biomedical samples are inherently complex and weak. The use of multivariate analysis can improve their applicability and extract the useful information that Raman spectroscopy can provide to biomedicine.

In this study two multivariate techniques: Principal Component Analysis (PCA) and Partial least square-discriminant analysis (PLS-DA) were performed over the pre-processed Raman spectra in order to evaluate the spectral differences between the cancerous cell lines studied and to develop a model allowing their discrimination and classification.

PCA operates in an unsupervised manner (no previous knowledge of the samples under study is provided) and finds an alternative set of coordinates, the principal components, (PCs) to reduce the dimensionality and complexity of the data set. All the spectra can then be explained in a much simpler fashion through a small number of PCs that accounts for the maximum variance in the data. In a PCA model, the matrix containing the set of spectra (X) is decomposed into two smaller matrices (the scores (T) and the loadings (P)): X = TP^T^+E where E is the residual explaining non-useful information that could not be explained by the multiplication of the scores and the loadings for each spectrum. By plotting the first Principal Components scores, relations between samples (grouping) are revealed. In addition, plotting loadings as a function of the wavenumbers reveal the most important diagnostic variables or regions in the spectra related with the differences found in the data set.

PLS-DA is a supervised classification method in which knowledge of the sample (in our case, malignant or benign phenotype) is included. PLS-DA employs the fundamental principle of PCA but further rotates the component (latent variables, LVs) by maximizing the covariance between the spectral variation and group affinity so that the LVs explain the diagnostically relevant variations rather than the most prominent variations in the spectral dataset. In this study, the performance of the PLS-DA diagnostic algorithm was validated using the venetian blinds cross validation methodology with eight data splits. The number of retained LVs was determined based on the minimal root mean square error of cross validation (RMSECV) curves, and finally six were taken.

Multivariate statistical analysis was performed using the PLS toolbox (Eigenvector Research, Wenatchee, WA) in the Matlab (Mathworks Inc., Natick, MA) programming environment. SPSS (Statistical Package for the Social Sciences) for Windows was used for the statistics of TFA and TUFA quantification. In all the analyses, differences were considered significant when student's “t” was lower than 0.05.

Before including Raman spectra in the multivariate statistical techniques, correct preprocessing must be performed. In this case, background subtraction was achieved with a Matlab and Labview algorithm [29], and then normalization under all Raman spectra was performed to correct for the different amplification in the signal. This normalization can be based on the fact that the spectral region used (the CH stretching region) can be considered as the total biomass present in our confocal volume [30].

### Real-time reverse transcription-PCR

Real-time reverse transcription-PCR (qRT-PCR) was performed with gene-specific fluorescent SYBR Green probes (Applied Biosystems, NY, USA) using a 7300 Real time PCR system detection Instrument and the associated software (Applied Biosystems), following the manufacturer's instructions. Primers were designed using Primer Express software (primer sequences are available on request). We calculated relative changes by the comparative C_T_ method using cyclophilin A as the reference gene. Each reaction was performed in triplicate.

## Results and Discussion

### The expression of lipid metabolic genes is correlated to the metastatic ability of cells

**Figure 1:**
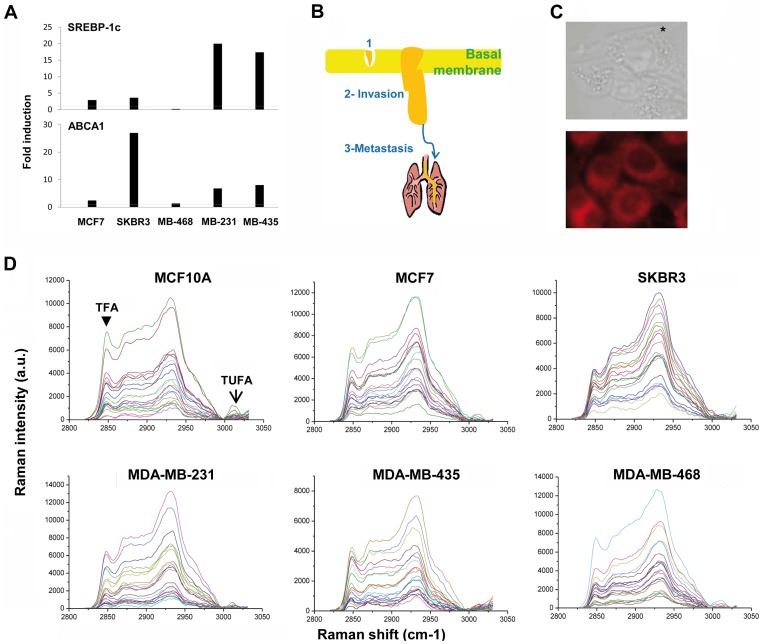
Variability in lipid metabolic genes expression and FA composition in MDA-MB-231, MDA-MB-435, MDA-MB-468, MCF7 and SKBR3 cell lines analysed by Raman microspectroscopy. A) The gene expression of SREBP-1c and ABCA1 were examined after 24 h treatment with the LXR agonist T0901317 2 µM compared to the basal conditions by RT and real-time PCR. The fold induction is represented over the pointed line. Cyclophilin A gene was used for normalization. B) Simplistic representation of the progression status of breast cancer cells used in the study: 1) MCF10A cells; 2) MCF7, SKBR3 and MDA-MB-468 and 3) MDA-MB-231 and MDA-MB-345. C) Above, brightfield image of MDA-MB-435 cells, with an asterisk indicating the position of the measurements in the cytoplasm. 60× magnification and 9 mW power were used. Down, fluorescence microscopy image of MDA-MB-435 cells stained with Nile red. 40× magnification was used. D) Measured raw Raman spectra of the cell lines where the axes are intensity (in arbitrary units) versus Raman shift (cm^−1^). MCF10A cells were also measured. The TFA (2845 cm^−1^) and TUFA (3015 cm^−1^) bands are indicated with the arrows in the first spectra.

The transcription factors SREBP-1c (transcriptional regulator sterol regulatory element-binding protein-1) and LXR (liver X receptor) maintain cholesterol homeostasis through complementary pathways of feedback inhibition and feed-forward activation [15], [31], [32]. To assess their coordinated action in the lipid phenotype of breast cancer cells, we explored the LXR pathways in a set of breast cancer cells according to their malignant phenotype including both non-metastatic and metastatic cells: MCF7, which expressed hormone receptors like luminal A tumors; SKBR3, a phenotype with amplifications of the ErbB2 oncogene; MDA-MB-468, p53 mutated cells with basal-like phenotype; and two different metastatic models: MDA-MB-435, with lung metastasis tropism, and MDA-MB-231 with bone metastasis tropism, both belonging to the basal-like phenotype (also called post-EMT cells) [33]. We analyzed the expression of SREBP-1c, gene target of LXR, and ABCA1, other direct LXR target gene involved in cell cholesterol export [19]. Twenty-four hours after treatment with 2 µM LXR agonist T0901317 (Figure 1A), the up regulation of SREBP-1c was evident in the metastatic cells MDA-MB-231 and MDA-MB-435 compared with non-metastatic cells: the transcriptional induction of SREBP-1c was 20 times higher in MDA-MB-231 and 17.4 times higher in MDA-MB-435. In contrast, treatment with the agonist produced only moderate increases in the expression of SREBP-1c in SKBR3 (2.9 times) and MCF7 (3.6 times), and a decrease in MDA-MB-468 cells. Moreover, the cholesterol related gene ABCA1 was increased in MDA-MB-231 (6.8 fold) and MDA-MB-435 (8 fold) and differently induced in non-metastatic cells (SKBR3 cells, 27 fold, MCF-7, 2.4 fold, and MDA-MB-468, 1.3 fold). These results showed the differences in regulation of lipid metabolism pathways in breast cancer cells.

Like nutritional control, neoplastic lipogenesis is controlled through the modulation of the expression and/or maturation status of the transcription factor SREBP-1c, a crucial intermediate of the pro- and anti-lipogenic actions of nutrients and hormones, which stimulates fatty acid synthase transcription in normal and malignant cells [32], [34]. In tumor cells, SREBP-1c expression and/or maturation is constitutively driven by the aberrant hyperactivation of these pathways in response to a variety of oncogenic changes, including overproduction of growth factors (GFs), ligand-dependent or independent hyperactivation of GF receptors (GFRs), and loss of function of components of the signalling cascade such as the phosphatase and tensin homologue (PTEN), a potent tumor suppressor [35], [36].

SKBR3 cells, a classical ErbB2 amplified model, responded against the LXR agonist with increased ABCA1 expression, different to that of MDA-MB-468 cells which have two populations with different degrees of EGFR expression [37], displaying low response against the agonist. It is well known that endogenous synthesized fatty acids increase the signal-to-noise ratio in the HER1/HER2-driven progression of human breast epithelial cells towards malignancy [13]. Malignant cells have devised a mechanism to subvert the normal pathways for feedback inhibition via the EGFRvIII and PI3K-dependent activation of SREBP-1c [15]. SKBR3 overexpressed SREBP-1c at basal levels by a factor of five with regard to MDA-MB-468 and metastatic cells; therefore, LXR might respond to excess cellular cholesterol by promoting ABCA1-dependent cholesterol efflux [31]. On the other hand, in normal cells, PI3K activation is tightly controlled by dephosphorylation of PIP3 by the phosphatase PTEN. Activity of the pathway is deregulated in cancer through a variety of mechanisms, including activating mutations in PI3K or PTEN loss [38], [39]. Indeed, the role of cholesterol metabolism in cancer pathogenesis and its association with EGFR/PI3K signaling has recently been described as a potential therapeutic target [15].

The inverse correlation between estrogen receptors in breast tumors and genes involved in lipid storage is well known [40]. Indeed, MCF7 cells had the lowest induction of SREBP-1c and ABCA1. In addition, the increased expression of SREBP-1c and ABCA1 in the estrogen negative metastatic cell lines indicated that both genes are functionally implicated in the most malignant phenotype. Therefore, the pathogenesis of metastasis may include the conjunction of both constitutive metabolic features: fatty acid synthesis and cholesterol cell content.

### The lipid phenotype characterized by Raman microspectroscopy

To explore the lipid phenotype associated to breast cancer malignancy we optimized the Raman microspectroscopy (RS) system to acquire Raman spectra in the range of 2820–3030 cm^−1^, where TFA (2845 cm^−1^) and TUFA (3015 cm^−1^) bands were located. In the analysis we included MCF10A cells as benign breast tumor cells, unable to spread outside the basal membrane, despite their basal-like phenotype (Figure 1B).

The cytoplasm lipids were measured by RS in a position near the nucleus and outside the endoplasmic reticulum area, where the Nile red staining showed major lipid concentration (see asterisk in Figure 1C). Each spectrum line in Figure 1D represents the Raman intensity versus the Raman shift measured in a single cell, and illustrates the cell variability inside each cell line. The bands corresponding to TFA and TUFA for individual cells were used to quantify the TFA and the TUFA average content in each cell line (Figure S1A and S1B). To obtain the relative quantities of unsaturated fatty acids (% UFA) in each cell line, which indicate the lipogenic phenotype, the values of individual cells followed by the average of cell lines were calculated (Figure S1C). Low but significant changes in the TFA bands intensities were found when the cell lines were compared with the MCF10A cells (Student's “t”<0.0009). The TFA content was clearly highest in MDA-MB-435 cells, followed by the MCF10A cells, and lowest in the SKBR3 cells. These results are in agreement with the increasing evidence that lipid accumulation is a hallmark of aggressive cancer cells, and is involved in the production of membranes for rapid cell proliferation [41].

TUFA bands intensities were only significantly lower in MDA-MB-468, SKBR3 and MCF7 cells when compared to MCF10A cells (Student's “t”<0.04). Moreover, no significant differences were found between MCF10A and the metastatic cells MDA-MB-231 (Student's “t” = 0.076) and MDA-MB-435 (Student's “t” = 0.661) with regard to TUFA. The unsaturation ratio, and not the TUFA value, is indicative of *de novo* lipogenesis and cell malignancy. For this reason, the percentage of unsaturated fatty acids in each cell line was calculated, but we did not observe significant differences due to the high dispersion in the individual cell values. The Student's “t” scores for the cells compared to MCF10A were: MDA-MB-435 = 0.179; MCF7 = 0.166; MDA-MB-231 = 0.323; MDA-MB-468 = 0.148 and SKBR3 = 0.139. Thus, the quantification of these two bands was not sensitive enough to differentiate benign from malignant breast cancer cells.

The total amount of lipids was analyzed with an alternative technique using Nile red staining (Figure S2). The red channel showed mainly the membrane phospholipids (hydrophilic lipids) and the green channel mainly the hydrophobic lipids (in yellow in the merge image), which accumulated in the typical cytoplasm storage vesicles derived from the endoplasmic reticulum compartment (called lipid droplets). They contain mainly esterified cholesterol and triglycerides [42]. The confocal images of lipids showed similar results to the RS quantifications with the exception of MDA-MB-468 cells, which showed the highest Nile red intensity. As expected, this technique was less informative than Raman for differentiating the cells. The green channel intensities labeling the lipid droplets did not correspond to the TUFA quantification obtained by Raman. MCF7 cells did not show lipid droplets, but their RS quantification was similar to that of SKBR3 and MDA-MB-468 cells. The RS results might also lead us to expect more droplets in MCF10A cells. The overestimation of unsaturated lipid content in MCF7 and MCF10A cells using RS may be due to differences in the lipid composition of the droplets [41].

The idea that exacerbated lipogenesis provides immortalized epithelial cells with a profound neoplastic growth and/or survival advantage over those that maintain physiological levels of endogenous fatty acid biosynthesis strongly suggests that some lipogenic enzymes may work as metabolic intermediates of oncogenesis by linking cellular anabolism and malignant transformation [43], [44]. Indeed, the level of fatty acid saturation indicative of *de novo* lipogenesis decreased when LNCaP prostate cancer cells were treated with soraphen A (a lipogenesis inhibitor) [17].

**Figure 2:**
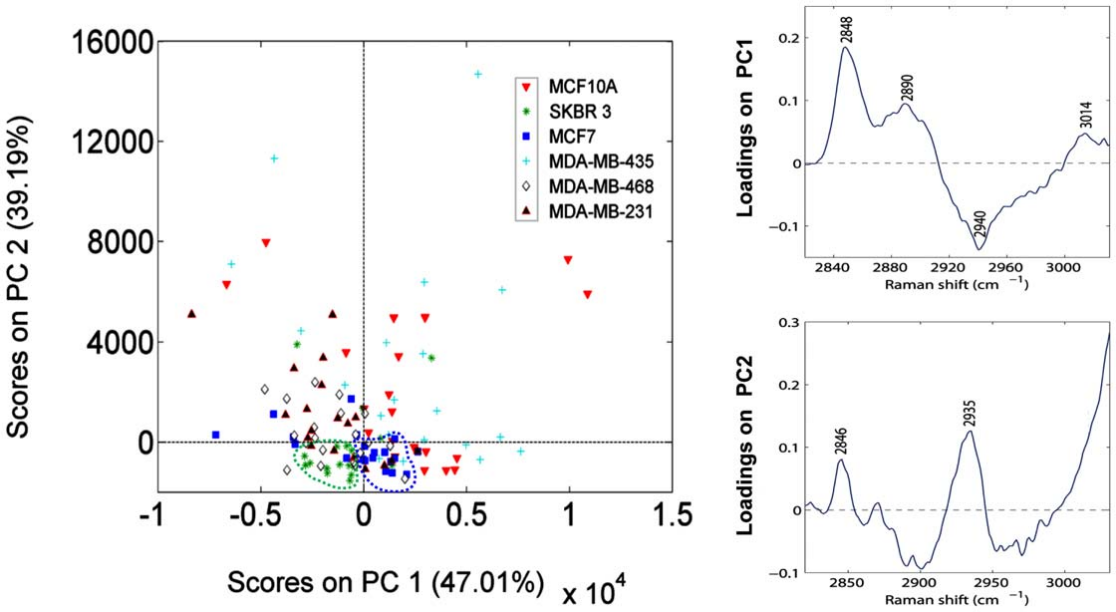
PCA scores showing the cell variability present inside each cell line and between the different cell lines. Illustration of PCA scores from MCF10A, MDA-MB-231, MDA-MB-435, MDA-MB-468, MCF7 and SKBR3 cell lines RS acquisition. SKBR3 cells are shown in the green circle and MCF7 cells in the blue circle. MDA-MB-435 and MCF10A cells are the most dispersed in the plot. On the right, the loading plots for each Principal Component, both related to fatty acid and protein content (TUFA: 3014 cm^−1^; protein and lipid: 2940 cm^−1^; TFA: 2871, 2890, 2846 and 2848 cm^−1^; -CH3: 2935 cm^−1^). Percentages in the score plots represent the variance accounted for each PC.

To improve the information obtained with the lipid phenotype measurements, we performed a PCA analysis using the 2820–3030 cm^−1^ spectral data to study the grouping and the homogeneity of the sample distribution (Figure 2). PC1 and PC2 scores accounted for 47% and 39% respectively of the total variance in the dataset. Raman band regions responsible for the PC1 score discrimination were 3014, 2890 and 2848 cm^−1^ (related to lipid content) and 2940 cm^−1^ (related to lipid and protein content). Raman band regions responsible for the PC2 score discrimination were 2846 cm^−1^ (TFA) and 2935 cm^−1^, associated with the chain end –CH3 [45].

MCF7 and SKBR3 cells were grouped in the low PC2 region and separated for high and low levels of the PC1 axis respectively. In contrast, other cell lines like MCF10A and MDA-MB-435 appear to be more heterogeneous, spreading through a larger area in the PC axes (Figure 2, left panel). Although Raman spectral region 2900 to 3100 cm^−1^ has been labeled the CH stretching region [45] and therefore, contains bands common for many biomolecules, we attempted to extract a hypothesis from the PCA score plot. As lipids were included in both PC1 and PC2 loadings, we interpret that SKBR3 and MCF7 had the lowest content. MDA-MB-231 and MDA-MB-468 cells had intermediate lipid content and MDA-MB-435 and MCF10A cells differed widely, though it was always high. Our interpretation of the PC1 loading was that it represents mainly TFA, and that lipid and protein cell content were inversely correlated, because we had lipid bands in positive and the 2940 cm^−1^ band (which includes both lipids and proteins) in negative. MCF7 and SKBR3 cells, with similar PC2 scores, had different PC1 values, suggesting different protein content, higher in SKBR3.

The most prominent band included in the PC2 loading was 2935 cm^−1^. No one substrate is clearly associated with the 2935 cm^−1^ band due to the fact that many biomolecules contain –CH3 side terminal groups. The 2846 cm^−1^ band, also included in PC2, corresponds to total fatty acids and we hypothesized a contribution of cholesterol and cholesterol esters in the 2935 cm^−1^ band, because it is a lipid with many –CH3 side terminal groups. We also observed differences in the ABCA1 gene expression, and differences in cholesterol have been associated to proliferation and migration of breast cancer cells [40], [41], suggesting the involvement of cholesterol in the malignant phenotype of the cells studied.

We analyzed the cholesterol content of the cell lines using filipin staining, which labels free (unesterified) cholesterol present in the cytosol and membranes of cells. We found differences in the content and distribution of free cholesterol between the cell lines. MDA-MB-231, MDA-MB-468 and MDA-MB-435 cells presented the highest cholesterol intensity, though with different distributions: in MDA-MB-231 and MDA-MB-468 it was accumulated in large cytoplasm spots, whereas MDA-MB-435 cells mainly presented cholesterol in the plasma membrane together with some smaller spots in the cytosol (Figure S3). The levels of cholesterol were also high in MCF10A but low in MCF7 cells. SKBR3 cells had higher cholesterol content than expected, given their localization in the PCA, but reinforcing our hypothesis that their high ABCA1 gene expression occurs in response to an excess in cholesterol content (see Figure 1A). Taken together, these results suggested that cholesterol might be involved in the lipid differences between metastatic and non-metastatic cells. Moreover, its contribution in the PC2 loading may also include cholesterol esters, not observed with filipin.

### The lipid profiling of breast cancer cells distinguishes metastatic ability from malignancy

**Figure 3:**
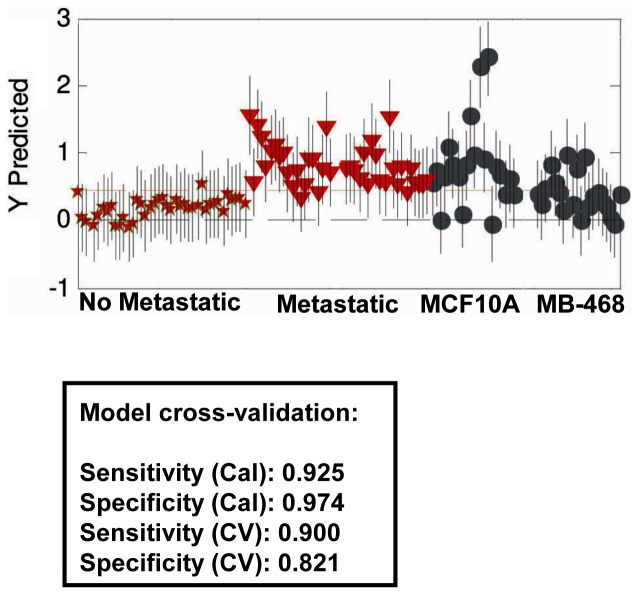
PLS-DA discriminative model using Raman microspectroscopy spectra of non-metastatic (SKBR3 and MCF7) and metastatic (MDA-MB-231 and MDA-MB-435) cell lines. PLSDA classification algorithm, in which non-metastatic cells are predicted with class 0 and metastatic cells with class 1. A threshold is assigned (red line) corresponding to the best specificity and sensitivity parameters that separate groups of cells. RMSECV is represented by the error bars. A sensitivity of 90% and a specificity of 82.1% were achieved. Once the model was built, MCF10A and MDA-MB-468 were included to predict their membership. Seventy-five per cent of MCF10A and 40% of MDA-MB-468 cells are related to the metastatic group.

In the second step, a PLS-DA was used to construct a classification model. This is a supervised method, meaning that prior knowledge of the class membership was included. First we built a classification algorithm to discriminate between non-metastatic non-basal-like (MCF7 and SKBR3) and metastatic basal-like (MDA-MB-231 and MDA-MB-435) cell lines. Then, in the ideal prediction model, the first group will have class 0 and the second one class 1 (Figure 3). The PLS-DA model was carried out over the pre-processed Raman spectra and a cross-validation was performed in order to check the strength of the algorithm to predict new samples. The method for cross-validation was venetian blinds w/10 splits and the errors for the prediction and cross validated model were RMSEC: 0.3 and RMSECV: 0.45 respectively, showing good stability for predicting new samples. A good discrimination between metastatic and non-metastatic cell lines was achieved with sensitivities and specificities of 92.5% and 97.4% for the calibration and 90% and 82.1% for the cross-validation respectively. These results showed good accuracy in discriminating metastatic ability of breast cancer cells, better than those reported for the Raman spectral window (2,800–3,100 cm^−1^) comparing benign disease and breast cancer tissue *in vivo* samples, which had specificity and sensitivity of 81.2 and 72.4, respectively [46].

We used the PLS-DA model to test the membership of MCF10A and MDA-MB-468 cells, which were not included in the groups (Figure 3). The result indicated that most of the MCF10A cells were very similar to the metastatic group. Seventy-five per cent of the MCF10A cells analyzed were predicted to belong to the metastatic class (above the threshold). The rest of the MCF10A cells were localised below the threshold. Following the same criteria eight of the twenty MDA-MB-468 cells analyzed (40%) were localized in the metastatic group and the rest in the non-metastatic group. The PLS-DA model showed that MCF10A and MDA-MB-468 cells had different basal-like phenotypes. These results suggested that the classification algorithm might discriminate two different basal-like phenotypes: MDA-MB-468 cells, common to a subgroup of MCF10A cells, and the MCF10A cells, common to MDA-MB-231 and MDA-MB-435 metastatic cells.

**Figure 4:**
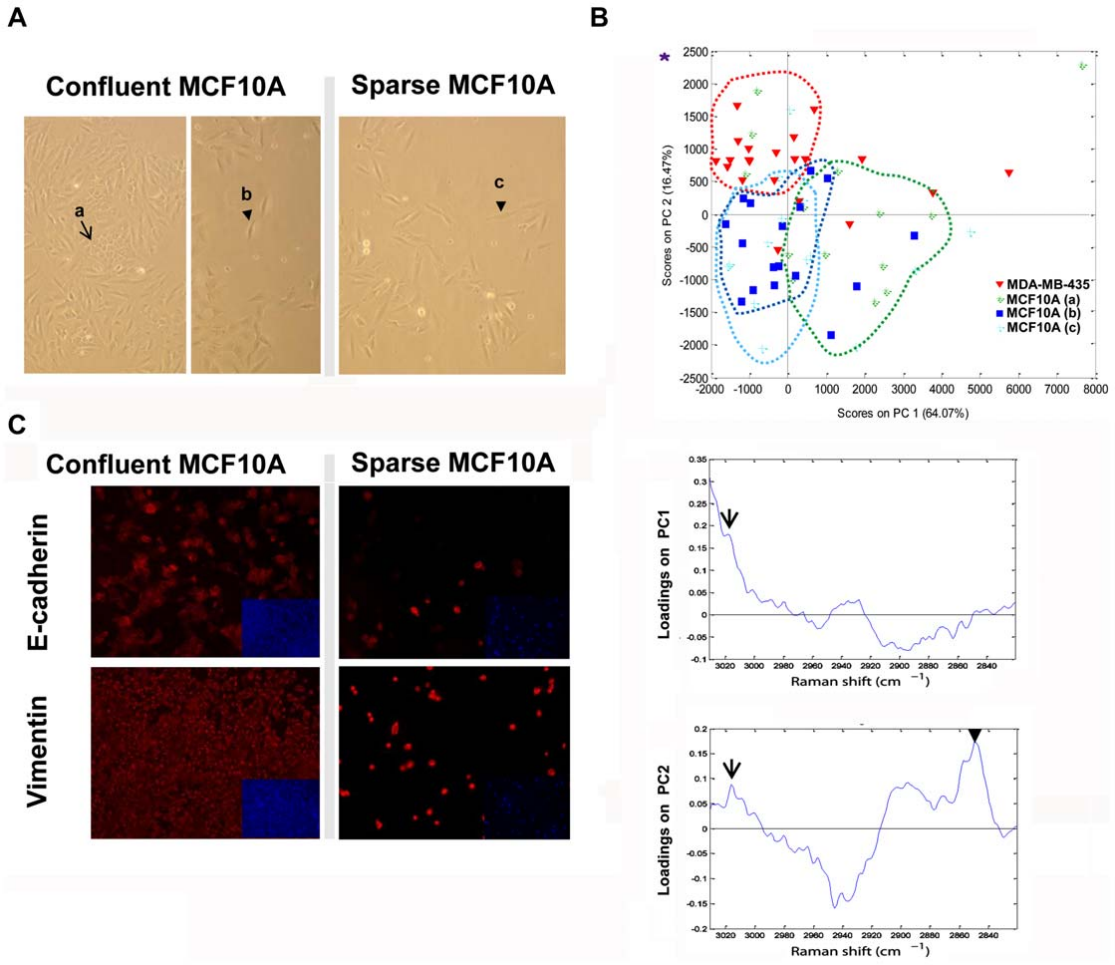
Raman microspectroscopy and PCA differentiate the MCF10A cells grown in confluent and sparse conditions. A) MCF10A microscopy images of the cells measured by RS in confluent and sparse conditions. Brightlight images were obtained with an inverted microscope and 10× magnification. Arrows indicate the different areas that were measured by Raman (a: confluent; b: separate cells grown at the edge of confluent cultures; c: sparse). B) PCA representation of MDA-MB-435 cells and MCF10A cells (grown in high confluence and in sparse conditions). PC 1 and 2 separate different groups of cell lines. MDA-MB-435 cells have higher PC2 scores, separated from the MCF10A. MCF10A grown in high confluence are displaced from the ones grown in sparse conditions showing higher PC1 scores. Asterisk indicates the localization of the “lipogenic phenotype” in the axis. PC1 and PC2 loadings are described down with the bands related to TFA (arrow head) and TUFA (arrow) indicated. The percentage means the variance accounted for each PC. C) Immunofluorescence images of E-cadherin and vimentin proteins in both MCF10A culture conditions. DAPI staining appears minimized inside each picture.

It has been described that MCF10A cells with basal-like phenotype, which present many features of mesenchymal cancer cell lines in sparse cultures, have intrinsic plasticity for undergoing EMT, transition present in the most aggressive breast tumors with a basal phenotype [28]. Since MCF10A cells were grown at low confluence, we hypothesized that the similarities between MCF10A and MDA-MB-435 cells in the PCA and between MCF10A and the metastatic cells group in the PLS-DA might be related to culture conditions. We performed Raman analysis in sparse and in dense MCF10A cultures, and in cells growing at the edge of dense cultures (Figure 4A). In the PCA (Figure 4B), PC1 had a prominent TUFA band contribution, and PC2 loading was formed by three lipid bands: 3014, 2890 and 2845 cm^−1^, and the 2935 cm^−1^ band with inverse correlation. The PCA scores plot clearly separated MCF10A and MDA-MB-435 cell lines: the phenotype of the MDA-MB-435 cells was characterized by higher lipid content (y axis) and lower TUFA (x axis). TUFA band intensity (PC1, x axis) also distinguished between the MCF10A subtypes, being lower in the sparse area and higher in the confluent area.

We also analyzed the EMT phenotype of MCF10A cells grown in sparse and confluent conditions. As expected, like MDA-MB-435, cells lost E-cadherin and expressed more vimentin in sparse conditions (Figure 4C) than in confluence. These results confirmed that the spectroscopical differences were secondary to phenotypic changes and correlated well with malignancy; clearly the degree of similarity between MDA-MB-435 and MCF10A cells depends strongly on the culture conditions of MCF10A.

**Figure 5:**
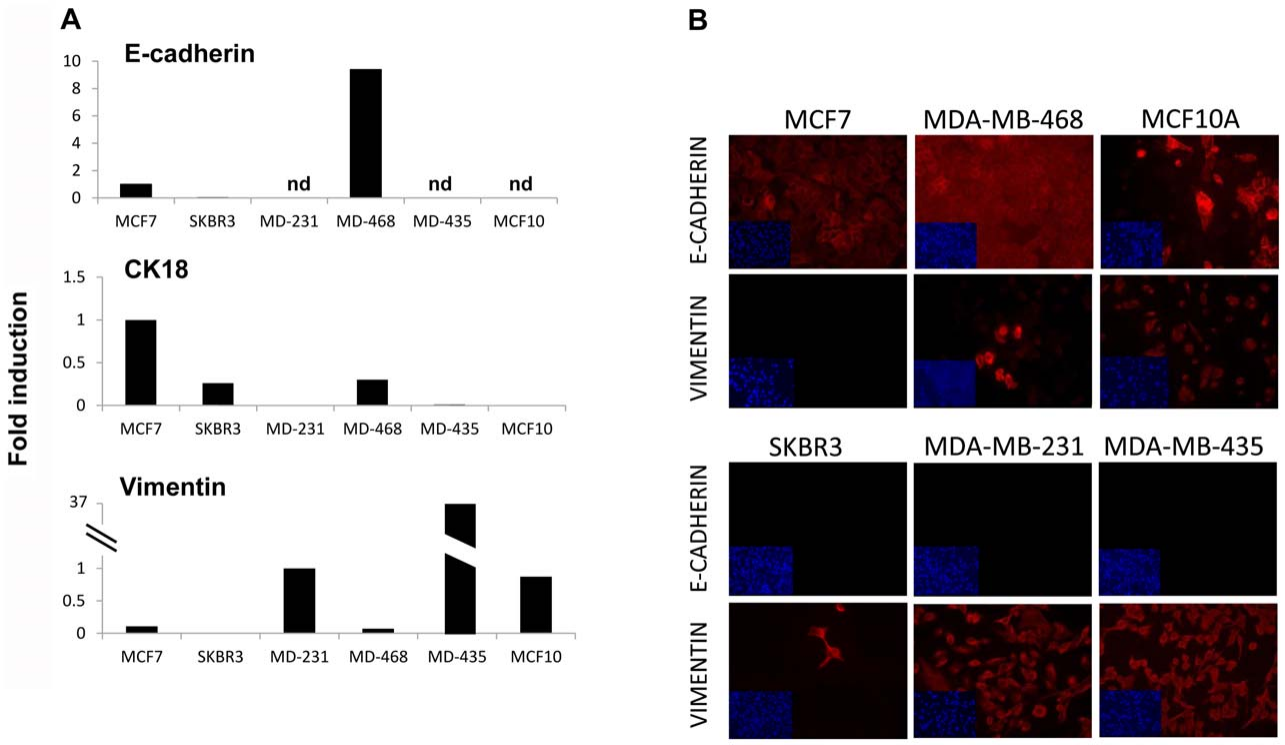
Epithelial and EMT marker gene expression in MCF7, SKBR3, MDA-MB-231, MDA-MB-468, MDA-MB-435 and MCF10A cells. A) The gene expression of epithelial cell markers (E-cadherin, cytokeratin 18) and the mesenchymal cell marker vimentin were examined by RT and real-time PCR using 200 ng of RNA. E-cadherin and CK18 are represented compared to the luminal MCF7 cell line expression and vimentin is represented compared to the MDA-MB-231 cell line. Cyclophilin A gene was used to normalize gene expression. B) Immunofluorescence staining of the epithelial E-cadherin and the mesenchymal vimentin markers in MCF7, MDA-MB-468, MCF10A, SKBR3, MDA-MB-231 and MDA-MB-435 cells. DAPI staining appears minimized in each picture. 40× magnification was used.

The expression in the set of breast cancer cells of E-cadherin, CK18 and vimentin at the mRNA level, and E-cadherin and vimentin at protein level as well, confirmed the close relationship between the lipid phenotype and the EMT process (Figure 5). MCF7 did not express vimentin protein and SKBR3 did so in less than 5% of the cells (Figure 5B), similar to MDA-MB-468 cells. These results suggested that in addition to metastatic ability the PLS-DA model discriminated cells with basal-like phenotype that undergo EMT (MCF10A) from basal-like cells with no EMT (MDA-MB-468).

The combination of multivariate statistical techniques applied to the Raman spectral data (PCA and PSL-DA analysis) provided a powerful quantitative method to discriminate cancer phenotypes. These mathematical methods used the whole range of the spectra for the differentiation of the cells. Our results suggest that the lipid phenotype of these cells is a signal of the proclivity to mesenchymal transition related to the high aggressiveness and metastatic spread [47]. EMT is an essential developmental process by which cells of epithelial origin lose epithelial characteristics and polarity, and acquire a mesenchymal phenotype with increased migratory behavior. Thus, the characterization of this functional phenotype of cancer cells with RS provides information on intercellular cell adhesion, down-regulation of epithelial markers, up-regulation of mesenchymal markers, acquisition of fibroblast-like (spindle) morphology with cytoskeleton reorganization, increase in motility, invasiveness, and metastatic capabilities [47]–[49]. The PSL-DA model described discriminates luminal or HER-2 overexpressing cells without EMT and post-EMT cells with a sensitivity of 90% and a specificity of 82.1%. Aggressive cells with basal phenotype (related to EMT plasticity) can also be differentiated, although it may be necessary to include other spectral regions to increase the sensitivity in the differentiation of metastatic and non-metastatic basal-like cell phenotypes. Recently, it has been reported that the analysis of human tumor gene expression profiles identifies triple negative breast cancer subtypes with an overall false-positive rates of 1.7%, 1.7%, and 0.9% for *ER*, *PR*, and *HER2*, respectively [50].

Breast cancer is a heterogeneous disease that includes a wide range of histological subtypes and a diversity of clinical behaviors and patient outcomes [51]. We used representative cell variants, including different phenotypes of breast cancer cells: estrogen receptor expression, ErbB2 amplification, p53 mutation and aggressive metastasic. The molecular and cellular characterization of their associated ‘lipid signatures’ by RS, combined with multivariate statistical analysis, is a promising technique for characterizing the malignant phenotype of breast cancer cells and might provide a helpful adjunct to gene-expression profiling or proteomics in the classification, diagnosis and prognosis of human cancers. Using different spectral ranges of RS, similar results have been obtained regarding the lower lipid content in SKBR3 compared to MDA-MB-231 and -435 cells [52]. These findings support the use of this technology in the study of the lipid phenotype of cells, with possibilities to be used in experimental tumors [46], [53] and in human samples to distinguish between ductal carcinoma *in situ* and invasive ductal carcinoma of the breast [54]. Serum samples have been used to discriminate between breast cancer patients and healthy individuals; the bands analyzed were statistically accepted as markers corresponding to proteins, polysaccharides and phospholipids [55]. Moreover, the identification of new spectral signatures expanding the RS window may offer more accurate classification of cells for diagnostic purposes, providing rapid, reagent-free and non-destructive alternatives for the analysis of tumor samples.

Raman spectroscopy has shown promise for use as a clinical tool for diagnosis of breast cancer. Optimization of spectral acquisition times and spatial resolution for clinical use is an area which needs further investigation. Studies of larger patient population samples will be needed to establish comparisons between spectral makers for breast cancer cells and pathological indicators that are used for current diagnosis. Moreover, improvements on current data analysis techniques, including the application of advanced data mining methods, along with novel preprocessing techniques will also be critical to introduce RS in the clinical practice.

## Conclusions

Raman spectroscopy is a promising technique in biomedical studies due to its non-invasive character and high specificity. The lipid phenotype associated to breast cancer malignancy belongs to Raman spectra adquired in the range of 2820–3030 cm^−1^, where TFA (2845 cm^−1^) and TUFA (3015 cm^−1^) bands were located. The combination of multivariate statistical techniques, which use the whole range of the spectra, applied to the Raman spectral data (PCA and PSL-DA analysis) provided a powerful quantitative method to discriminate cancer phenotypes. In addition, an algorithm to differentiate metastatic from non metastatic and non basal phenotype breast cancer cells was design using PLS-DA, with 90% sensitivity and 82.1% specificity. Our results suggest that the lipid phenotype of these cells is a signal of the proclivity to mesenchymal transition related to the high aggressiveness and metastatic spread, then the identification of new spectral signatures expanding the RS window may offer more accurate classification of cells for diagnostic purposes.

## Supporting Information

Figure S1 **Analysis of the lipid content in the breast cancer cell lines using Raman microspectroscopy.** A) Total fatty acid (TFA) and B) total unsaturated fatty acid (TUFA) Raman band intensity average in the cell lines is represented in arbitrary units. C) Relative unsaturated fatty acid content is represented as %. The average was calculated with the individual cell ratio values. The lines and the p values (student's “t”) indicate the significance between bars compared to the MCF10A values.
(TIF)

Figure S2 **Analysis of the lipid content in the breast cancer cell lines using Nile red staining and confocal microscopy.** Cells were fixed in 4% PFA and treated with Nile red (1 µg/ml) for 1 h at room temperature and analysed as indicated in material and methods. Hydrophilic fatty acids, mainly phospholipids, are seen in the red channel. Hydrophobic fatty acids, mainly cholesterol esters and triglycerides, are seen in the merge image in yellow. DAPI staining labels the nuclei. 40× magnification was used.
(TIF)

Figure S3 **Analysis of the cholesterol content in MCF7, SKBR3, MDA-MB-231, MDA-MB-468, MDA-MB-435 and MCF10A cells with filipin staining and fluorescence microscopy.** Cells were fixed in 4% PFA and treated with filipin (50 µg/ml) for 2 h at room temperature and analyzed as indicated in material and methods. Filipin labels free cholesterol present in the membranes (arrow) and in the cytosol (arrow head). 40× magnification was used.
(TIF)
