# Supplementary figures and images for: The Lipid Phenotype of Breast Cancer Cells Characterized by Raman Microspectroscopy: Towards a Stratification of Malignancy
Source: PLoS One. 2012 Oct 17;7(10):e46456. doi: 10.1371/journal.pone.0046456 (PMC3474759; doi:10.1371/journal.pone.0046456)

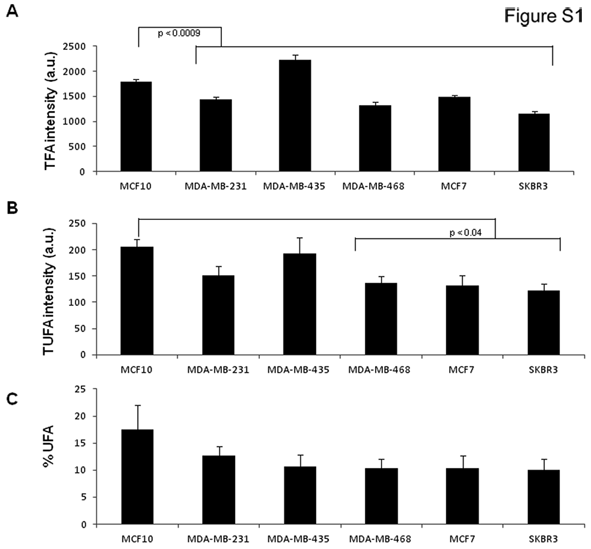

Supplement: Figure S1 — Analysis of the lipid content in the breast cancer cell lines using Raman microspectroscopy. A) Total fatty acid (TFA) and B) total unsaturated fatty acid (TUFA) Raman band intensity average in the cell lines is represented in arbitrary units. C) Relative unsaturated fatty acid content is represented as %. The average was calculated with the individual cell ratio values. The lines and the p values (student's “t”) indicate the significance between bars compared to the MCF10A values. (TIF) [file pone.0046456.s001.tif]

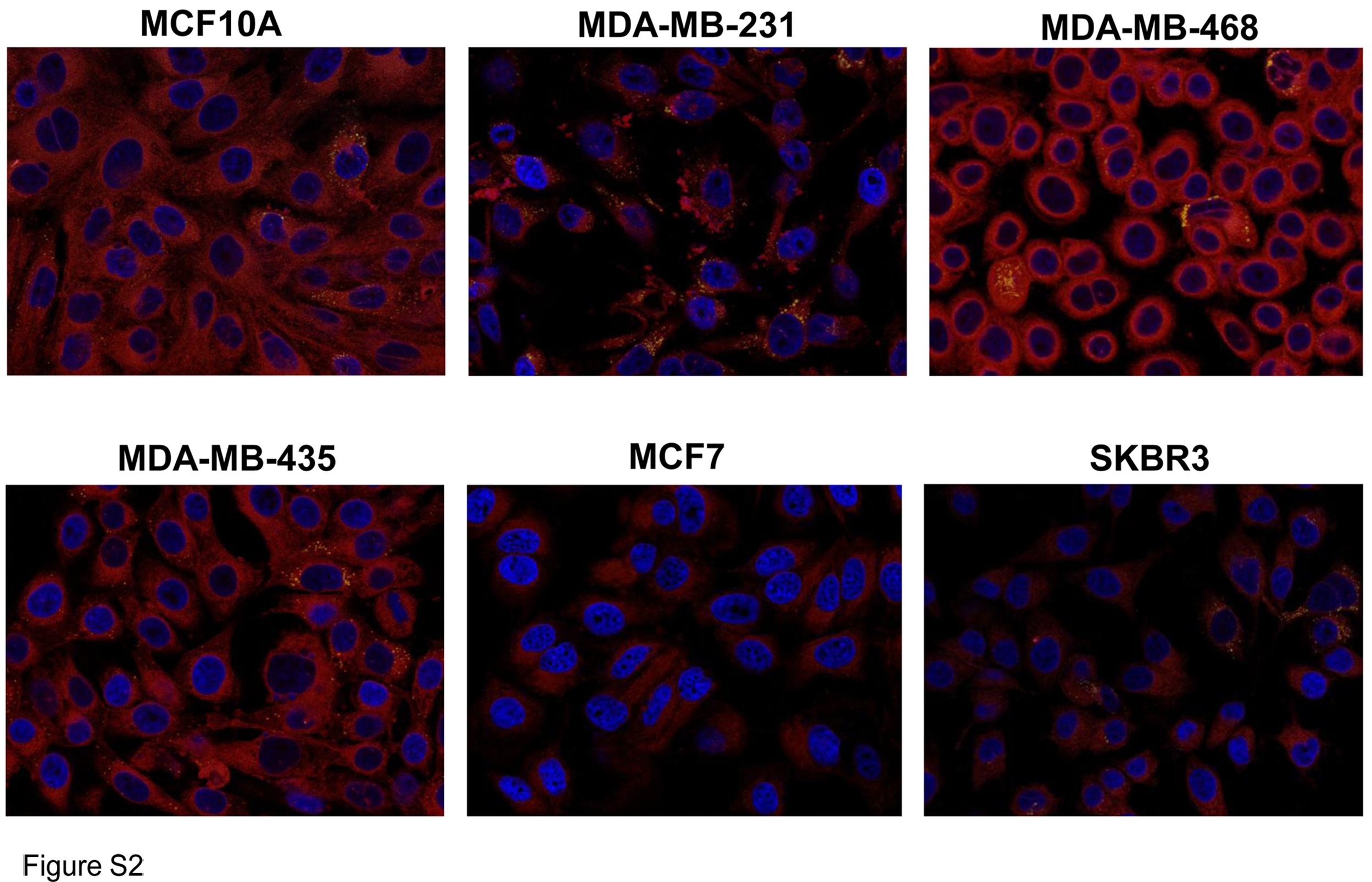

Supplement: Figure S2 — Analysis of the lipid content in the breast cancer cell lines using Nile red staining and confocal microscopy. Cells were fixed in 4% PFA and treated with Nile red (1 µg/ml) for 1 h at room temperature and analysed as indicated in material and methods. Hydrophilic fatty acids, mainly phospholipids, are seen in the red channel. Hydrophobic fatty acids, mainly cholesterol esters and triglycerides, are seen in the merge image in yellow. DAPI staining labels the nuclei. 40× magnification was used. (TIF) [file pone.0046456.s002.tif]

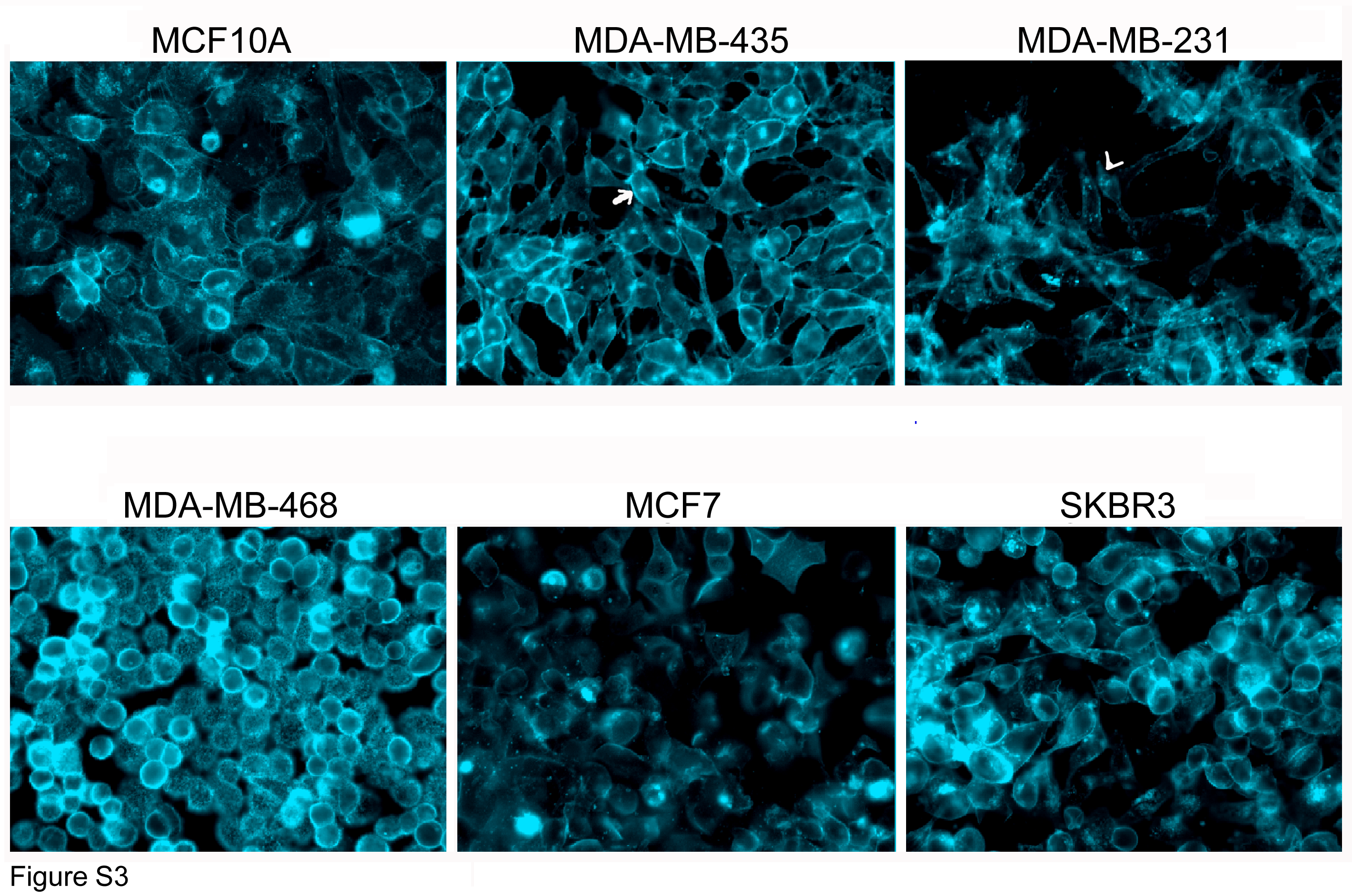

Supplement: Figure S3 — Analysis of the cholesterol content in MCF7, SKBR3, MDA-MB-231, MDA-MB-468, MDA-MB-435 and MCF10A cells with filipin staining and fluorescence microscopy. Cells were fixed in 4% PFA and treated with filipin (50 µg/ml) for 2 h at room temperature and analyzed as indicated in material and methods. Filipin labels free cholesterol present in the membranes (arrow) and in the cytosol (arrow head). 40× magnification was used. (TIF) [file pone.0046456.s003.tif]
